# Comparison of Blood Pressure and Kidney Markers between Adolescent Former Preterm Infants and Term Controls

**DOI:** 10.3390/children7090141

**Published:** 2020-09-17

**Authors:** Eveline Staub, Natalie Urfer-Maurer, Sakari Lemola, Lorenz Risch, Katrina S. Evers, Tatjana Welzel, Marc Pfister

**Affiliations:** 1 Department of Neonatology, Royal North Shore Hospital, St Leonards, NSW 2065, Australia; 2 Department of Neonatology, University of Basel Children’s Hospital, 4056 Basel, Switzerland; Katrina.Evers@ukbb.ch; 3 Department of Psychology, University of Basel, 4056 Basel, Switzerland; natalie.urfer.maurer@gmail.com; 4 Centre for Early Intervention, Department of Education, 4001 Basel-Stadt, Switzerland; 5 Department of Psychology, University of Bielefeld, 33615 Bielefeld, Germany; S.Lemola@warwick.ac.uk; 6 Department of Psychology, University of Warwick, Coventry CV4 7AL, UK; 7 Labormedizinische Zentren Dr Risch, 9490 Vaduz, Liechtenstein; lorenz.risch@risch.ch; 8 Department of Pediatric Pharmacology and Pharmacometrics, University of Basel Children’s Hospital, 4056 Basel, Switzerland; tatjana.welzel@ukbb.ch (T.W.); ma.pfister@unibas.ch (M.P.)

**Keywords:** adolescent, follow-up studies, hypertension, kidney, prematurity

## Abstract

Background: Preterm infants are at an increased risk of developing hypertension and chronic kidney disease later in life. No recommendations exist for blood pressure (BP) and renal follow up for these patients. Aim: To compare BP and serum and urinary kidney markers between preterm-born adolescents and term-born controls. Methods: BP measurements in 51 preterm-born (≤32 weeks gestational age) and 82 term-born adolescents at the age of 10–15 years were conducted. Stepwise regression analysis explored the association between BP and participant characteristics. Kidney markers measured in the serum and urine were creatinine, neutrophil gelatinase-associated lipocalin (NGAL), and uromodulin. Kidney markers measured in the serum were cystatin C, beta-2 microglobulin, and beta trace protein. Results: Systolic BP was significantly higher in preterm boys compared with term boys, but not in girls, and low birth weight was associated with higher BP in boys. In the preterm group, maternal hypertension/preeclampsia and adolescent height were associated with higher systolic BP. Serum creatinine and NGAL were significantly higher in the preterm group. Conclusions: Our study confirms an inverse sex-dependant relationship between birth weight and BP at adolescent age. The higher serum creatinine and NGAL in the preterm group may indicate that premature birth affects kidney function in the long term.

## 1. Introduction

Infants born at a very young gestational age (GA) and a very low birth weight (VLBW) are at risk for developing renal and cardiovascular diseases when they grow up to adolescents and young adults [1]. Large studies have shown that birth weight (BW) and GA are inversely associated with an increased risk of developing arterial hypertension and chronic kidney disease (CKD) [2]. According to the theory of developmental origins of adult disease, the kidneys are subject to multiple pre- and postnatal influences which permanently alter structure and function in the long term. Maternal risk factors, preterm birth, and low birth weight result in low nephron numbers, and low nephron endowment, in turn, makes the kidneys more vulnerable to additional damage through a variety of pathophysiological factors [3]. While close neurodevelopmental follow up of former VLBW infants is the standard of care, risk assessment of impaired kidney function and cardiovascular disease in childhood and adolescence is not. Routine measurement of blood pressure (BP) is the obvious choice for screening for arterial hypertension. However, it is unclear whether the assessment of albuminuria and the estimated glomerular filtration rate (eGFR) from serum creatinine capture the early decline in kidney function in preterm-born patients who typically have reduced renal mass with hypertrophied, hyperfiltrating individual glomeruli [4]. Alternative kidney marker candidates to detect decreasing kidney function earlier or more reliably are the low-molecular-weight proteins cystatin C (CysC), β-2 microglobulin (B2M) and β-trace protein (BTP), which are filtered freely in glomeruli. CysC and B2M are then reabsorbed and metabolized in the proximal tubuli, while BTP is excreted in urine. CysC does not appear to discriminate between former preterm-born adolescents and term controls [5], while B2M and BTP have not been explored in this context. Neutrophil gelatinase-associated lipocalin (NGAL) is expressed in the tubuli and considered a marker for tubular damage in acute kidney injury (AKI), but is also upregulated during evolving CKD [6]. Increased urinary NGAL levels in adults with former VLBW compared to those born at normal BW have been reported [7], but no data are available on serum NGAL in this population. Uromodulin (Uromod), previously referred to as Tamm–Horsfall protein, is expressed exclusively in tubular cells of the thick ascending Henle limb and protects tubular cells during infection and stone formation [8]. Uromod has not been investigated for the adolescent age bracket after preterm birth.

This study compared BP in a cohort of adolescents born preterm with term controls to test our hypothesis that preterm birth leads to increased BP. We explored pre-, peri-, and postnatal risk factors associated with higher BP; and we present profiles of established and newer glomerular and tubular kidney markers, some of which have not been described in this population: creatinine (Crea), CysC, NGAL, B2M, BTP, and Uromod in serum and Crea, NGAL, and Uromod in urine.

## 2. Materials and Methods

### 2.1. Participants

This was a cross-sectional analysis embedded in the longitudinal cohort of the Basel Study of Preterm Children (BSPC). Previous reports of the first and second study waves have described recruitment procedures [9,10]. In brief, for the preterm group, preterm infants born at ≤32 + 0 weeks GA admitted to the University of Basel Children’s Hospital Neonatal Intensive Care Unit (NICU) between June 2001 and December 2005 were approached for recruitment at the age of 10 to 15 years. Inclusion criteria were (1) no severe developmental delays, (2) residence within a radius of 100 km of the birth hospital, and (3) sufficient knowledge of German for parents to give written consent. For the term group, age-and sex matched controls born at ≥37 weeks gestation and born in the same period as the preterm born study participants were recruited from official birth notifications at the same ages. For each participant, parents gave written informed consent to participate in one or both of the study components (home visit and/or lab visit, see below). The local ethics review committee approved the study protocol (reference number EK122/11), and the study was performed according to the ethical standards of the Declaration of Helsinki.

### 2.2. Measurements during the Home Visit

Study participants were visited at home. Weight was measured on digital calibrated scales. Height was measured with a folding rule. Body mass index (BMI) was calculated as BMI = weight [kg]/(height [m])^2^. A BMI ≥ 85th and <95th centile for age and sex defined overweight, while a BMI ≥ 95th centile for age and sex defined obesity.

Upper arm circumference (measured mid-way between the acromion and olecranon with a non-elastic tap measure) on the non-dominant arm determined the BP cuff size. After a seated rest period of 10 min, BP was measured twice with one minute between measurements (using an Aponorm Professional 47034 oscillometric device). The average systolic and diastolic BP from the two measurements were used for data analysis. Elevated BP was defined according to the 2017 American Academy of Pediatrics (AAP) guidelines as BP > 90th and <95th centile and hypertension-level BP was defined as BP ≥ 95th centile for age, sex, and height centiles [11]. Study participants collected mid-stream urine at the time of first urine void in the morning after BP measurement. Urine samples were then transported on ice to the pathology laboratory and frozen at −20 °C without prior centrifugation within 2 h after collection. After completion of home visits, urine samples were sent in one batch on dried ice to the analysis facility. After thawing of samples, laboratory analysis of urine Crea, NGAL, and Uromod was performed in one batch in fully automated analysers according to methods specified in supplementary Table S1 (in Supplementary Materials).

### 2.3. Measurement during Lab Visit

Study participants presented to the ambulatory study facility at the University of Basel Children’s Hospital for blood collection. Topical anaesthetic cream was applied one hour prior to collection of 10 mL of blood into EDTA vacutainers via direct venepuncture. Blood samples were centrifuged immediately and then frozen at −20 °C. After all lab visits had been completed, blood samples were sent in one batch on dried ice to the analysis facility. After thawing of samples, laboratory analysis of serum Crea, CysC, BTP, B2M, NGAL, and Uromod was performed in one batch in fully automated analysers according to methods specified in Table S1 (in Supplementary Materials). eGFR was calculated using the combined serum Crea and CysC quadratic formula [12].

### 2.4. Data Collection

To identify factors associated with BP, we obtained pre-, peri-and postnatal data for each study participant born preterm from discharge summaries of their NICU admission. These included pregnancy complications and treatments (assisted conception, smoking during pregnancy, gestational diabetes, pregnancy-induced hypertension or preeclampsia or HELLP syndrome (syndrome consisting of haemolysis, elevated liver enzymes, and a low platelet count), (complete or incomplete course of) antenatal steroid administration); delivery details (mode of delivery, Apgar scores at 1 and 5 min, arterial cord pH); and neonatal details (GA at birth, BW, z-score for BW, duration of respiratory support (combined days supported by invasive mechanical ventilation, continuous positive end-expiratory pressure, and humidified high flow nasal cannula), diagnosis of bronchopulmonary dysplasia (defined as the requirement of oxygen at 28 days of age plus need for either oxygen or positive pressure at 36 weeks postmenstrual age), necrotizing enterocolitis (any stage according to Bell staging criteria), retinopathy of prematurity (any degree), intraventricular haemorrhage (any degree), persistent ductus arteriosus and its treatment with non-steroidal anti-inflammatory agents, hypotension (as a diagnosis in discharge summary, i.e., no indication of absolute BP value available or whether treatment was required), antibiotic treatment (no further specification of antibiotic class available in discharge summary), and treatment with postnatal steroids. For the term group, only information from official birth notifications was available, including GA at birth, BW, and z-score for BW, but no further details on pre-or perinatal details. Small for gestational age (SGA) was defined as a z-score for birth weight of below −1.28.

### 2.5. Analyses

Statistical analysis was performed using the *R* statistical software package (version 3.6.0, RRID:SCR_001905). Descriptive data were displayed using means (±standard deviations) for normally distributed variables and medians (with interquartile ranges (IQRs)) for other continuous variables. Differences in characteristics between the preterm and term groups were assessed using Wilcoxon‘s rank sum test and Student’s *t*-test depending on the distribution of variables. We then built multivariable regression models to assess associations between systolic or diastolic BP and participant characteristics using the Akaike information criterion (AIC) for step-wise model selection. The first model was performed over the whole cohort, using the characteristics available for both the preterm and term groups (GA, BW, age and anthropometry as assessment); the regression analyses were performed over the whole cohort. For this model, BW and GA were dichotomized as <2500 g or >2500 g and preterm or term. As this analysis of BP differences demonstrated a significant difference in preterm and term boys, but not in girls, this analysis was extended to explore sex differences by building separate models for each sex. The second model included additional characteristics only available for the preterm group (i.e., details of diagnosis and treatment during the NICU admission). GA and BW were used as continuous variables. To avoid overfitting, variables with less than 10 events per variable and group were not used in the regression analysis. Due to the small sample size, this analysis was not performed separately for each sex.

For kidney markers, analysis was restricted to descriptive statistics due to small sample size.

## 3. Results

### 3.1. Participant Characteristics

**Figure 1:**
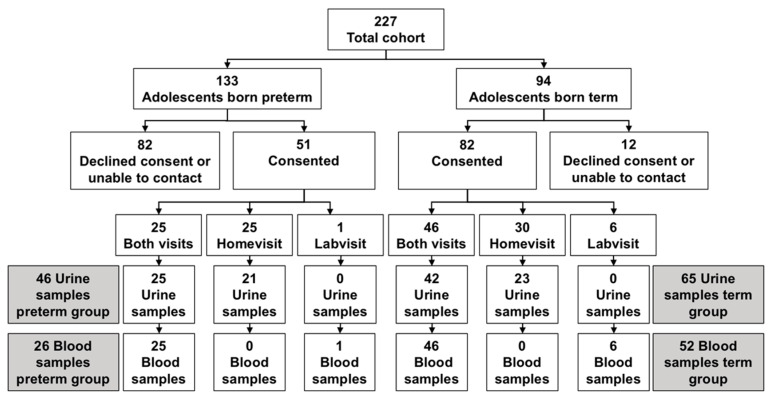
Participant flow diagram of recruitment and procuring of urine and blood samples from the original cohort.

Fifty-one former preterm-born adolescents and 82 term-born controls consented to either both or one of the study components. Forty-six urine samples and 26 blood samples were obtained from study participants in the preterm group, and 65 urine samples and 52 blood samples were obtained from the term group (Figure 1). The adolescents who did not consent to study participation or who could not be contacted for recruitment had similar BW and GA values at birth to the study participants. There were minor variable differences between the subgroups within the preterm- and term-born participants who did or did not provide either urine or blood samples or both (Table S2 in Supplementary Materials).

Table 1 describes the general characteristics for both the preterm and term groups by sex. Naturally, GA and BW were significantly lower for the preterm group compared with the term group. The Z-score for BW and the prevalence of small for GA status were similar in both groups, as was sex distribution. Participants in both groups were of similar ages and had similar anthropometric measures (weight, height, BMI) at the time of assessment.

For the preterm group, Table 2 presents the details of their initial neonatal admission including morbidities and treatments. Approximately one-fifth of pregnancies were complicated by either hypertension in pregnancy or a disorder on the preeclampsia spectrum. The prevalence of gestational diabetes was low. Approximately two-thirds of participants in this group received steroids for lung maturation prior to preterm delivery. The median duration of respiratory support was less than a week. The prevalence of severe neonatal morbidities (bronchopulmonary dysplasia, necrotizing enterocolitis, intraventricular haemorrhage, and retinopathy of prematurity) was low. Slightly over half of the infants received antibiotic treatment. Less than 10% received postnatal steroids.

### 3.2. Blood Pressure

Systolic and diastolic BP values were similar in the preterm group and the term group, with a similar prevalence of elevated and hypertension-level BP (Table 1). However, systolic BP was on average 5.1 mmHg higher in males born preterm compared with systolic BP values of males born at term. Systolic and diastolic BP values were similar in the preterm and term females.

Regression models for all girls showed that age and height at assessment were significant predictors of systolic BP (Table 3). None of the predictors were significant predictors of diastolic BP. For the boys, weight at the time of BP assessment was a significant predictor for both systolic and diastolic BP and low birth weight for diastolic BP. When the model was restricted to the preterm group, two factors predicted systolic BP: maternal hypertension or preeclampsia and height at the time of the BP assessment (Table 4). The model accounted for 48% of the variance in systolic BP. For diastolic BP, GA and antenatal steroid cover were significant predictors. The model accounted for 36% of the variance in diastolic BP.

### 3.3. Glomerular and Tubular Kidney Markers

Serum Crea and NGAL were significantly higher in the preterm group compared to serum Crea and NGAL in the term group (Table 5). eGFR was similar in both groups. None of the study participants in either group had eGFR < 60 mL/min/1.73 m^2^, which is the functional criteria for CKD if present for longer than 3 months, as defined in the KDIGO (Kidney Disease: Improving Global Outcomes) guidelines [13]. All other kidney markers in the serum and urine were similar in both groups.

## 4. Discussion

In this cohort, the average systolic BP was higher in preterm-born adolescent boys compared with term-born adolescent boys. Only body weight at the time of BP assessment was predictive of systolic and diastolic BP in boys, while age and body height at BP assessment were predictive of systolic BP in girls. For the group of preterm-born study participants, we found associations between systolic BP and maternal hypertension or preeclampsia and body height at the time of BP assessment, and between diastolic BP and GA and antenatal steroid cover. The group of pretermborn adolescents had higher serum Crea and NGAL values compared with the term-born adolescents.

### 4.1. Blood Pressure

Our finding of higher systolic BP in preterm-born boys contrasts with other studies, where preterm-born women were found to be at a higher risk for developing hypertension, but the association between preterm birth and high BP was present in both sexes [14]. There is an increasing acknowledgement of sex differences in health trajectories after preterm birth, and overall, males appear to be more vulnerable to adverse outcomes [15]. Genetic and immunological factors and influences of sex hormones are discussed as causes for the sex differences. Androgens are prominently involved in BP regulation and lead to higher BP values in adult men compared with women [16]. Preterm-born boys have higher androgen levels than term-born boys from early infancy, leading to potential long-term implications including higher BP and increased risk for adverse cardiovascular events [17]. We did not record puberty status in our cohort; with ages between 10 and 15 years, the participants would range from still pre-pubertal to the later puberty stage.

The association of lower BW and GA with increased BP later in life has been described previously [18]. A similar mechanism to the Brenner hypothesis with glomerular hyperfiltration of fewer nephrons in addition to other peri- and postnatal factors are postulated as being associated with offspring born to mothers with preeclampsia [19]. Animal data suggest that the effect of antenatal steroids on later BP is likely to be multifactorial with a basis of lower nephron endowment [20], although reports of the long-term effects in humans are contradictory [21,22].

### 4.2. Glomerular and Tubular Kidney Markers

Our study found a higher serum Crea level in the preterm-born adolescents, but no difference in eGFR. The bulk of evidence has identified preterm birth and/or LBW or VLBW as risk factors for lower eGFR by the time patients reach young adulthood [5], although some studies did not find a difference in renal function [23]. Serum Crea has a smaller intra-individual variability than measured GFR [24]. Some evidence suggests that it performs well in detecting even small changes in kidney function in early-stage renal disease [25]. Other markers of kidney function might have similar potential to identify early CKD. CysC and BTP promise to reliably predict GFR and CKD progression, possibly better than serum Crea [6,25]. CysC has been explored in childhood or adolescence after preterm birth and found not to discriminate between preterm-born and term-born individuals [5]. We are among the first to report BTP levels in this population. These have the potential to act as a good biomarker in a state of hyperfiltration (of the oligonephritic kidney) [26].

Serum and urine NGAL have been used to diagnose acute kidney injury and predict the progression of CKD [6]. Increased urinary NGAL levels in former ELBW adults compared with those of term-born controls have been reported before [7], but our finding of elevated serum NGAL in this population is new. Considering the tubular origin, elevated serum NGAL levels could indicate chronic tubular damage and decreased tubular function. Some authors hypothesize that elevated NGAL indicates its production in chronically injured tubular cells and therefore makes it a reliable marker for ongoing kidney damage [27]. While the focus of chronic kidney damage in former preterm infants has mostly been around the oligonephritic state and subsequent hyperfiltration [4], there is a paucity of evidence describing the state of the tubular epithelium in this group of patients beyond the neonatal age.

Equally, serum and urine B2M and Uromod have not been reported before in the adolescent age bracket after preterm birth. B2M reflects GFR independent of sex and body weight in adults [28] but is primarily used to assess tubular kidney function [29]. Uromod has the potential to not only predict the progression of CKD but to actually reflect the number of intact nephrons, because patients with atrophied or low numbers of tubular cells have been shown to have low urine and serum Uromod values [30]. While Uromod in our cohort was similar in the preterm and term groups, it could have the potential to identify former born adolescents with reduced renal mass who are at increased risk of developing chronic kidney dysfunction in a larger population.

### 4.3. Limitations

Our study has a few limitations. Firstly, study participants were recruited for both BP measurements through kidney function testing and psychological testing, requiring a sufficient level of verbal communication. Therefore, adolescents with severe cognitive impairment were not invited to participate in the study. Children with severe developmental sequelae after preterm birth may have suffered from the most severe neonatal morbidities, with presumably more severe impacts on kidney development and function. This recruitment bias may have led to an attenuation of the difference in BP and kidney markers in comparison with the control group. Secondly, we did not have access to neonatal data for the term-born control group (other than GA and BW) or recent medical history and drug exposure in both groups. Therefore, we were unable to exclude any study participant with a history of kidney disease or other illness or drug exposure impacting renal function or BP. However, none of the adolescents had eGFR < 70 mL/min/m^2^. Additionally, the differences between the study participants who provided blood and/or urine samples and those who did not provide either of the samples may have led to a degree of selection bias. It is challenging to infer the influence of these inconsistent inter-group differences on the study results, particularly with the small number of participants in some of the groups.

Thirdly, we did not measure proteinuria or albuminuria. Albuminuria as a marker for glomerular filtration barrier injury or dysfunction has been incorporated into the defining criteria of CKD in the KDIGO definition [13]. This precluded us from comparing albuminuria with the other kidney markers. Also, urine and blood samples were not collected on the same day; therefore, direct comparisons between urine and serum values of the same biomarker were not feasible. Finally, the sample size was relatively small and particularly analyses on biomarkers may have been insufficiently powered. Studies with larger samples will allow to draw firmer conclusions.

## 5. Conclusions

Preterm birth presents a multitude of risk factors for elevated BP in adolescence. The males in our cohort appeared to be more vulnerable to long-term cardiovascular sequelae after preterm birth. To date, no kidney marker has been shown to reliably identify declining renal health years after the interruption of kidney development by preterm birth and associated neonatal morbidities. Large scale prospective studies are required for serial follow up of children born at low GA and VLBW to validate individual kidney markers in urine or serum and identify those at increased risk for CKD.

## Figures and Tables

**Table 1:** Characteristics of former preterm-born study participants and term-born control study participants, as complete groups and by sex.

	Preterm (*n* = 51)	Female (*n* = 25)	Male (*n* = 26)	Term (*n* = 82)	Female (*n* = 35)	Male (*n* = 47)
Gestational age at birth ^†^ (wks + d)	31 + 0 (2 + 6) *	31 + 2 (4 + 3) *	30 + 4 (2 + 3) *	39 + 6 (2 + 5)	39 + 0 (2 + 0)	40 + 0 (2 + 1)
Birth weight ^†^ (g)	1360 (532) *	1360 (682) *	1390 (490) *	3250 (555)	3200 (435)	3380 (635)
Z-score birth weight ^†^	0.08 (0.81)	0.08 (1.12)	0.10 (0.62)	0.10 (1.32)	0.07 (0.72)	0.09 (1.64)
SGA	5 (9.8%)	3 (12%)	2 (7.7%)	8 (9.8%)	2 (3.7%)	6 (12.8%)
Age at assessment ^†^ (years)	12.3 (1.87)	12.2 (1.68)	12.3 (1.46)	12.1 (1.20)	11.8 (1.24)	12.2 (1.09)
Weight at assessment ^†^ (kg)	44.2 (19.9)	45.0 (15.8)	43.8 (23.9)	43.4 (14.6)	43.9 (14.4)	43.1 (15.4)
Height at assessment ^†^ (cm)	155.0 (17.7)	154.0 (18.0)	156.0 (20.4)	154.0 (11.5)	153.5 (13.7)	154.0 (10.5)
BMI at assessment ^†^ (kg/m^2^)	19.0 (4.4)	19.1 (4.0)	18.4 (6.0)	18.3 (3.8)	18.3 (4.2)	18.1 (3.7)
Overweight	3 (6%)	0	3 (12%)	8 (10%)	4 (11%)	4 (9%)
Obesity	5 (10%)	3 (12 %)	2 (8%)	3 (4%)	0	3 (6%)
Systolic blood pressure ^‡^ (mmHg)	109.4 (±8.36)	107.6 (±8.56)	111.5 (±7.78) ^#^	106.2 (±10.42)	105.7 (±10.79)	106.4 (±10.50)
Elevated systolic blood pressure	1 (2%)	1 (4%)	0	5 (6%)	3 (9%)	2 (4%)
Hypertension-level systolic blood pressure	4 (8%)	2 (8%)	2 (8%)	4 (5%)	2 (6%)	2 (4%)
Diastolic blood pressure ^‡^ (mmHg)	66.9 (±5.88)	66.4 (±6.25)	67.5 (±5.49)	65.2 (±6.17)	66.1 (±6.20)	64.6 (±6.13)
Elevated diastolic blood pressure	2 (4%)	2 (8%)	0	2 (2%)	2 (6%)	0
Hypertension-level diastolic blood pressure	0	0	0	0	0	0

Data are **^†^** median (IQR), **^‡^** mean (±SD) or n (%); wks = weeks; d = days; SGA = small for gestational age, defined as birth weight z-score <−1.28 for sex; BMI = body mass index; Overweight defined as BMI between 85th and 95th centiles for age and sex, obesity defined as BMI > 95th centile for age and sex; * *p* < 0.05 in preterm-born group (complete and separated by sex) compared to term-born group; **^#^** *p* < 0.05 in preterm-born males compared to term-born males.

**Table 2:** Details of pre-, peri-, and postnatal morbidities and treatments for the study participants born preterm.

	All (*n* = 51)
Prenatal details	
Multiple pregnancy	16 (31%)
Fertility treatment	7 (14%)
Gestational diabetes	1 (2%)
Pregnancy induced hypertension or preeclampsia or HELLP	10 (19%)
Antenatal steroids	32 (63%)
Smoking	6 (12%)
Perinatal details	
Vaginal delivery	4 (8%)
Apgar 1 min	6 (2)
Apgar 5 min	8 (1)
Arterial cord pH	7.32 (0.08)
Morbidities and details of NICU admission	
Length of neonatal hospital stay (d)	40 (24.5)
Days of respiratory support	3 (6.5)
Bronchopulmonary dysplasia	3 (6%)
Necrotizing enterocolitis	1 (2%)
Retinopathy of prematurity	1 (2%)
Intraventricular haemorrhage	2 (4%)
Patent ductus arteriosus, of which treated	9 (17%), 4
Hypotension	4 (8%)
Antibiotic treatment	28 (55%)
Postnatal steroids	4 (8%)

Data are median (IQR) or n (%); HELLP = syndrome consisting of haemolysis, elevated liver enzymes, low platelet count; NICU = Neonatal Intensive Care Unit

**Table 3:** Regression model for systolic and diastolic blood pressure for the whole cohort (preterm-born and control-term born study participants) by sex.

	Systolic Blood Pressure				Diastolic Blood Pressure			
	Girls		Boys		Girls		Boys	
	RC	*p*-Value	RC	*p*-Value	RC	*p*-Value	RC	*p*-Value
Birth weight category (<2500 g or >2500 g)	−3.15	0.18	−4.45	0.046			−3.20	0.034
Z-score birth weight	−2.20	0.08						
Age at assessment	−4.62	0.002						
Weight at assessment			0.43	<0.001			0.13	0.037
Height at assessment	0.69	<0.001			0.13	0.08		
R-square (adjusted)	0.21		0.29		0.04		0.10	
*p*-value for model	0.003		<0.001		0.09		0.011	

RC = estimate of regression coefficient. Stepwise regression model with AIC selection of model of best fit.

**Table 4:** Regression model for systolic and diastolic blood pressure in relation to pre-, peri-, and postnatal factors for preterm-born study participants.

	Systolic Blood Pressure		Diastolic Blood Pressure	
	RC	*p*-Value	RC	*p*-Value
Birth weight	−0.006	0.14		
Z-score birth weight				
Gestational age	0.17	0.15	0.21	<0.001
Male sex				
*Antenatal factors*				
Antenatal steroids			−3.73	0.017
Hypertension/ preeclampsia/HELLP	9.56	0.03		
Postnatal factors				
Arterial cord pH	0.25	0.05		
Days of respiratory support				
Factors at the time of study assessment				
Age at assessment	−1.98	0.11		
Height at assessment	0.58	<0.001		
R square (adjusted)	0.48		0.36	
*p*-value for model	<0.001		<0.001	

HELLP = syndrome consisting of haemolysis, elevated liver enzymes, low platelet count); RC = estimate of regression coefficient. Stepwise regression model with AIC selection of model of best fit. Variables with <10 events per group were excluded.

**Table 5:** Renal biomarkers in serum and urine in preterm-born and term-born control study participants.

	Preterm	Term Control	*p*-Value
Serum	*n* = 26	*n* = 52	
Creatinine (µmol/L)	61 (14)	55 (9.5)	0.024
Cystatin C (mg/L)	0.87 (0.16)	0.89 (0.17)	0.97
eGFR (mL/min/1.73m^2^)	90.4 (13.2)	96.2 (8.8)	0.10
NGAL (ng/L)	73.5 (21.8)	58.0 (25.0)	0.005
Beta-2 Microglobulin (mg/L)	1.6 (0.4)	1.6 (0.3)	0.95
Beta-trace Protein (mg/L)	0.60 (0.12)	0.58 (0.13)	0.35
Uromodulin (ng/mL)	194.3 (111)	197.3 (206)	0.70
Urine	*n* = 46	*n* = 65	
NGAL/Creatinine (ng/mg)	4.50 (3.57)	4.30 (4.25)	0.62
Uromodulin/Creatinine (ng/mg)	2.19 (2.17)	2.62 (3.16)	0.13

Data are medians (IQRs). Serum and urine Creatinine were analysed by the enzymatic method. eGFR = estimated glomerular filtration rate; Calculated by combined serum Creatinine and Cystatin C quadratic formula [12].

## Supplementary Materials

The following are available online at https://www.mdpi.com/2227-9067/7/9/141/s1, Table S1: Methods for analyzing renal biomarkers in serum and urine. Table S2: Characteristics of subgroups within the preterm born and term born group according to which, if any, samples were provided by the study participants.
