# Supplementary material for: Comparison of Blood Pressure and Kidney Markers between Adolescent Former Preterm Infants and Term Controls
Source: Children (Basel). 2020 Sep 17;7(9):141. doi: 10.3390/children7090141 (PMC7552693; doi:10.3390/children7090141)
Supplement: Supplementary file 1 [file children-07-00141-s001.pdf]

Supplementary Table S1. Methods for analysing renal biomarkers in serum and urine

**Analyte**	**Analyser**	**Measurement principle**	**Reagent manufacturer**	**Sample volume / Test (µl)**	**International Standardization**
Creatinine (Crea) enzymatic	Cobas Roche	Photometry	Roche Diagnostics	6	IDMS
Cystatin C (CysC)	Cobas Roche	Immuno-Turbidimetry	Roche Diagnostics	2	ERM-DA471/IFCC
β-trace protein (BTP)	Siemens BN II analyser	Nephelometry	Siemens Diagnostics	35	No
β_2-microglobulin (B2M)	Cobas Roche	Immuno-Turbidimetry	Roche Diagnostics	2	No
Neutrophil gelatinase associated lipocalin (NGAL)	Cobas Roche	Immuno-Turbidimetry	BioPorto	10	No
Uromodulin (Uromod)	DSX, Dynex Technologies	ELISA	Euroimmun	10	No

Supplementary table S2. Characteristics of subgroups within the preterm born and term born group according to which, if any, samples were provided by the study participants.

	**Preterm group** (n=51)				**Term group** (n=82)			
	Urine & blood samples (n=25)	Urine sample only (n=21)	Blood sample only (n=1)	No sample (n=4)	Urine & blood samples (n=42)	Urine sample only (n=23)	Blood sample only (n=10)	No sample (n=7)
Female	48%	45%	0%	80%	50%	26%	50%	43%
Gestational age (wks + d)	30+0	31+1	29+4	30+1	40+0	40+4	38+0	39+0
Birth weight (g)	1440	1485	1215	990	3310	3315	3055	3250
Systolic blood pressure (mmHg)	108.5	109.8	138	106	109	106	106	102
Diastolic blood pressure (mmHg)	65	69.5	76	64.5	65	64.5	62	65
Age at assessment (y)	12.4	12.2	11.4	13.5	11.8	12.4	12.2	12.0
Weight at assessment (kg)	47.5	38.2	35.5	53.5	42.1	39.7	48.3	51.0
BMI at assessment (kg/m2)	19.0	17.85	16.43	19.42	17.82	16.75	20.01	20.0

Data are median.
